# Referral patterns of GIST patients: data from a nationwide study

**DOI:** 10.2340/1651-226X.2024.23722

**Published:** 2024-02-14

**Authors:** Evelyne Roets, Nikki S. Ijzerman, Vincent K.Y. Ho, Ingrid M.E. Desar, Anna K.L. Reyners, Hans Gelderblom, Dirk J. Grünhagen, Boudewijn van Etten, Winan J. van Houdt, Winette T.A. van der Graaf, Neeltje Steeghs

**Affiliations:** a Department of Medical Oncology, The Netherlands Cancer Institute, Amsterdam, the Netherlands; b Department of Medical Oncology, Erasmus MC Cancer Institute, Erasmus University Medical Center, Rotterdam, the Netherlands; c Department of Research and Development, Netherlands Comprehensive Cancer Organisation (IKNL), Utrecht, the Netherlands; d Department of Medical Oncology, Radboud University Medical Center, Nijmegen, the Netherlands; e Department of Medical Oncology, University of Groningen, University Medical Center Groningen, Groningen, the Netherlands; f Department of Medical Oncology, Leiden University Medical Center, Leiden, the Netherlands; g Department of Surgical Oncology, Erasmus MC Cancer Institute, Erasmus University Medical Center, Rotterdam, the Netherlands; h University of Groningen, University Medical Center Groningen, Department of Surgical Oncology and gastrointestinal surgery, Groningen, the Netherlands; i Department of Surgical Oncology, The Netherlands Cancer Institute, Amsterdam, the Netherlands

**Keywords:** Gastrointestinal stromal tumor, GIST, registry, reference center, systemic treatment, mutation analysis

## Abstract

**Background:**

This study compares the characteristics, referral and treatment patterns and overall survival (OS) of gastrointestinal stromal tumor (GIST) patients treated in reference and non-reference centers in the Netherlands.

**Patients and methods:**

This retrospective cohort study on patients diagnosed between 2016 and 2019, utilises data from the Netherlands Cancer Registry and the Dutch Nationwide Pathology Database. Patients were categorized into two groups: patients diagnosed in or referred to reference centers and patients diagnosed in non-reference centers without referral.

**Results:**

This study included 1,550 GIST patients with a median age of 67.0 in reference and 68.0 years in non-reference centers. Eighty-seven per cent of patients were diagnosed in non-reference centers, of which 36.5% (493/1,352) were referred to a reference center. Referral rates were higher for high-risk (62.2% [74/119]) and metastatic patients (67.2% [90/134]). Mutation analysis was performed in 96.9% and 87.6% of these cases in reference and in non-reference centers (*p* < 0.01), respectively. Systemic therapy was given in reference centers versus non-reference in 89.5% versus 82.0% (*p* < 0.01) of high-risk and in 94.1% versus 65.9% (*p* < 0.01) of metastatic patients, respectively. The proportion of positive resection margins and tumor rupture did not differ between reference and non-reference centers. Median OS was not reached.

**Conclusion:**

A substantial amount of metastatic GIST patients in non-reference centers did not receive systemic treatment. This might be due to valid reasons. However, optimisation of the referral strategy of GIST patients in the Netherlands could benefit patients. Further research is needed to explore reasons for not starting systemic treatment in metastatic GIST patients.

## Introduction

Gastrointestinal stromal tumors (GIST) are the most common mesenchymal tumors of the gastrointestinal (GI) tract and represent approximately 1%–2% of primary GI cancers [1]. Incidence ranges between 10 and 15 cases per million per year and therefore GIST is a rare disease [2]. The Netherlands national guidelines state that patients should be referred or discussed with a reference center before starting treatment [3]. According to the report of the Society of all medical specialists clinically active in Oncology in the Netherlands (SONCOS), a sarcoma reference center should facilitate an experienced multidisciplinary sarcoma team, discussing at least 100 new sarcoma patients per year [3]. For GIST, there are 5 GIST reference centers constituting the Dutch GIST consortium (DGC) which prospectively collects detailed data of all GIST patients treated or followed-up in these centers since 2009. All other centers are defined as non-reference centers.

Treatment of GIST patients is based on the European Society of Medical Oncology (ESMO) guidelines [4]. Mutation testing and central review of pathology are incorporated in the ESMO guidelines since 2008 and 2010, respectively. The standard treatment for localized GIST >2 cm is complete surgical resection [4, 5]. High-risk GISTs as defined by the AFIP-Miettinen criteria have an indication to receive (neo)adjuvant imatinib [1]. Patients with localized inoperable GIST or metastatic GIST are treated with imatinib or other tyrosine kinase inhibitors (TKIs) [6].

Compliance to the ESMO guidelines in the Netherlands was previously investigated in smaller patient populations [7, 8]. No studies evaluated the real-life outcomes of GIST patients treated in non-reference centers. The primary objectives of this study are to compare characteristics, referral and treatment patterns between patients in GIST reference centers and non-reference centers in the Netherlands. Secondary objectives are to evaluate the rate of positive resection margins and tumor rupture of patients operated in reference centers and in non-reference centers and to compare the overall survival (OS) of patients with a high-risk GIST or metastatic GIST between both groups.

## Patients and methods

### Patients and study design

In the Netherlands, data of all cancer patients are collected in a national cancer registry (NCR), which is maintained by the Netherlands Comprehensive Cancer Organisation (IKNL). For each center, clinical data are extracted from the medical records and entered in the registry 6 to 9 months after diagnosis by trained data managers. Survival status is updated yearly. In the current study, only patients diagnosed between 2016 and 2019 were included, because data on mitotic rate, mutation status and treatment center (i.e. GIST reference center or non-reference center) were only systematically collected since 2016. Data acquisition was approved by the supervisory committee of the NCR, the local ethics committee of the Dutch Nationwide Pathology Databank (Palga) and the scientific committee of the DGC.

### Variables of interest

Demographic data, including age, year of diagnosis, gender, survival status, location of diagnosis and treatment center (i.e reference or non-reference center) were collected from the NCR. Performance status, comorbidity or patients’ preference regarding treatment were not registered. Patients were subdivided into two groups: patients diagnosed in a reference center or referred to a reference center for treatment or discussion at the multidisciplinary tumor board (MTB), and patients diagnosed in a non-reference center without referral to a reference center. Tumor-specific data, such as location, stage (localized or metastatic), size, mitotic rate (number of mitoses/50 High Power Fields), margin status (negative [R0], microscopically positive [R1], macroscopically positive [R2]) and intraoperative tumor rupture were retrieved from the NCR. Tumor rupture was determined according to the findings in the surgery report. Mutational status was derived from the Palga database and matched to the data obtained from the NCR database for all GISTs >2 cm according to the ESMO guidelines [9]. Standard mutational testing was performed for *KIT* and *PDGFRA*. Based on tumor location, size and mitotic index, the risk classification according to the AFIP-Miettinen criteria could be determined [1]. Tumor rupture was not considered in the risk assessment. The NCR registers treatments received in the first 6–9 months after diagnosis (imatinib or other TKI). The systemic treatment setting (i.e. [neo]adjuvant or palliative) was deduced from the sequence of provided treatments as registered in the NCR.

### Statistical analysis

Patient and tumor characteristics were described using descriptive statistics. Comparative analyses for categorical and continuous variables were done using the Chi-squared tests or Fisher’s exact test (for cells with < 5 counts), and the Mann-Whitney U test, respectively. Follow-up time was defined as the period between diagnosis and death or last date of follow-up. In patients with localized GIST, survival analysis was only performed for patients with a high-risk GIST. Survival curves were estimated using the Kaplan-Meier method. For patients with localized GIST, multivariable logistic regression analysis with tumor location and size as covariates was performed to investigate differences in the rate of R1/R2 resections and the rate of tumor rupture between patients operated in reference centers and in non-reference centers. Patients with unknown intent of surgery were excluded from the analysis regarding resection margins. A two-sided *p*-value of 0.05 was considered statistically significant. Statistical analyses were performed using IBM SPSS Statistics 27.

## Results

### Referral patterns, tumor characteristics and mutation analysis rate

**Figure 1:**
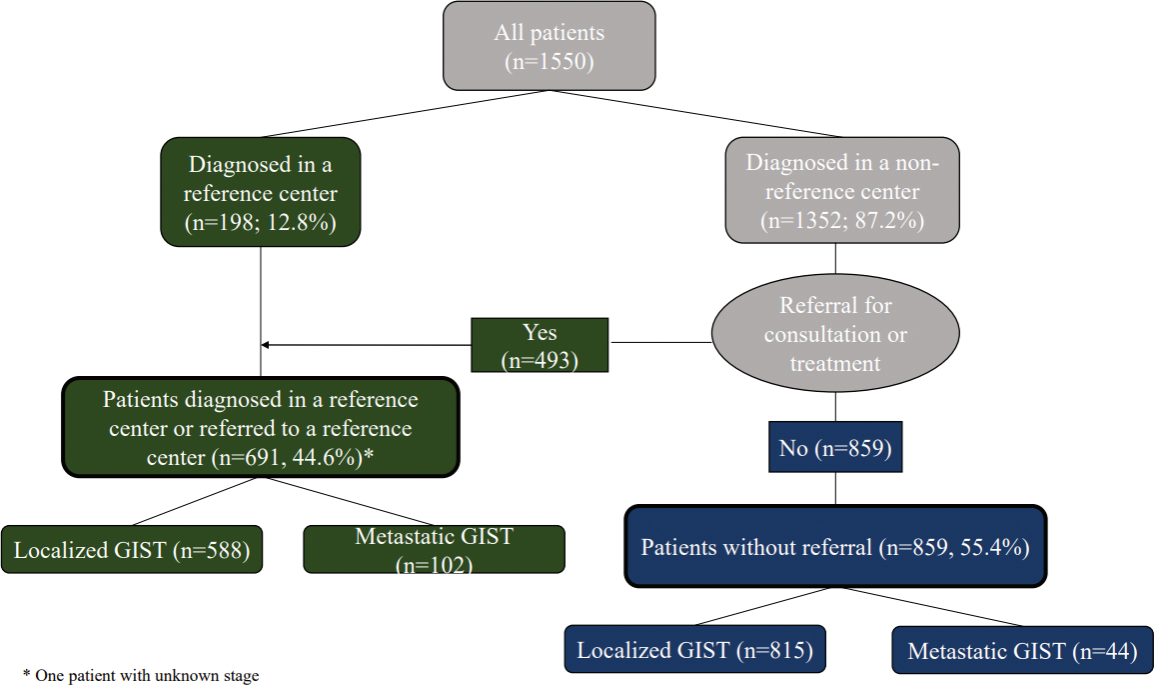
Referral patterns of GIST patients.

Between January 2016 and December 2019, 1,550 patients were diagnosed with GIST and registered in the NCR. Referral patterns of Dutch GIST patients are summarized in Figure 1. Almost two thirds of patients diagnosed in a non-reference center were not referred to a reference center upon diagnosis. The majority of patients with metastatic GIST (69.9%) were diagnosed in a reference center or referred to a reference center.

Patient and tumor characteristics for the total cohort and for the subgroup of patients with a metastatic GIST or a high-risk GIST are shown in Tables 1 and 2. Patients in non-reference centers more often had smaller tumors compared to patients in reference centers (*p* < 0.01). For both groups, the most prevalent tumor locations were the stomach and the small bowel (reference center: 66.1% and 18.4% resp.; non-reference center: 70.9% and 17.2% resp.). Compared to patients in non-reference centers, patients in reference centers more often had extra-gastric GIST (31.8% vs. 26.9%, *p* = 0.01). Patients with a high-risk GIST or metastatic GIST in non-reference centers were significantly older compared to patients in reference centers (70 years vs. 65 years, *p* < 0.01).

**Table 1:** Characteristics of Dutch patients with a GIST diagnosis in 2016–2019. Comparison between patients in reference centers and in non-reference centers. *N* (%) or median (range).

	Reference center (*n* = 691)	Non-reference center (*n* = 859)	Total (*n* = 1550)	*p*
**Median age at diagnosis (years)**	67.0 (19–94)	68.0 (28–95)	68.0 (19–95)	< 0.01
**Sex**				
Male	367 (53.1)	410 (47.7)	777 (50.1)	0.04
Female	324 (46.9)	449 (52.3)	773 (49.9)	
**Tumor stage**				
Localized GIST	588 (85.1)	815 (94.9)	1,403 (90.5)	< 0.01
Metastatic GIST	102 (14.8)	44 (5.1)	146 (9.4)	
Unknown	1 (0.1)	0 (0.0)	1 (0.06)	
**T stage**				
T0	3 (0.4)	5 (0.6)	8 (0.5)	< 0.01
T1 ≤ 2 cm	104 (15.1)	243 (28.3)	347 (22.4)	
T2 > 2 cm and ≤ 5 cm	221 (32.0)	345 (40.2)	566 (36.5)	
T3 > 5 cm and ≤ 10 cm	194 (28.1)	182 (21.2)	376 (24.3)	
T4 > 10 cm	158 (22.9)	65 (7.6)	223 (14.4)	
Tx (unknown)	11 (1.6)	19 (2.2)	30 (1.9)	
**Location**				
Stomach	457 (66.1)	609 (70.9)	1,066 (68.8)	0.01
Esophagus	11 (1.6)	16 (1.9)	27 (1.7)	
Small bowel	127 (18.4)	148 (17.2)	275 (17.7)	
Colon	10 (1.4)	16 (1.9)	26 (1.7)	
Rectum	32 (4.6)	14 (1.6)	46 (3.0)	
Duodenum	37 (5.4)	30 (3.5)	67 (4.3)	
Other^a^	3 (0.4)	7 (0.8)	10 (0.6)	
Unknown	14 (2.0)	19 (2.2)	33 (2.1)	
**Mutation analysis performed^b^**				
Yes	489 (86.4)	232 (39.3)	721 (62.4)	< 0.01
No	77 (13.6)	358 (60.7)	435 (28.0)	

a Peritoneum or pancreas.

b Mutation analysis is only reported for patients with a successful linkage between the dataset of the national cancer registry (NCR) and the Dutch Nationwide Pathology Databank (Palga) and only for patients with a primary GIST ≥2 cm or a metastatic GIST (1,156/1,550 patients).

**Table 2:** Characteristics of Dutch patients with a high-risk GIST (according to the AFIP-Miettinen criteria) or a metastatic GIST diagnosed in 2016–2019. Comparison between patients in reference centers and in non-reference centers. *N* (%) or median (range).

	Reference center (*n* = 193)	Non-reference center (*n* = 89)	Total (*n* = 282)	*p*
**Median age at diagnosis (years)**	65.0 (23–89)	70.0 (38–88)	67.0 (23–66)	< 0.01
**Sex**				0.33
Male	116 (60.1)	48 (53.9)	164 (58.2)	
Female	77 (39.9)	41 (46.1)	118 (41.8)	
**Location**				0.17
Stomach	101 (52.3)	51 (57.3)	152 (53.9)	
Esophagus	0 (0.0)	1 (1.1)	1 (0.4)	
Small bowel	52 (26.9)	23 (25.8)	75 (26.6)	
Colon	5 (2.6)	1 (1.1)	6 (2.1)	
Rectum	10 (5.2)	3 (3.4)	13 (4.6)	
Duodenum	14 (7.3)	1 (1.1)	15 (5.3)	
Other^a^	1 (0.5)	1 (1.1)	2 (0.7)	
Unknown	10 (5.2)	8 (9.0)	18 (6.4)	
**Mutation analysis performed^b^**				< 0.01
Yes	184 (96.9)	78 (87.6)	262 (93.0)	
No	6 (3.2)	11 (12.4)	17 (6.0)	

a Peritoneum.

b Mutation analysis rate is only reported for patients with a successful linkage between the dataset of the national cancer registry (NCR) and the Dutch Nationwide Pathology Databank (Palga) (279/282 patients).

The linkage between the NCR dataset and the Palga dataset was successful for 1,537/1,550 patients. Overall, mutation analysis was performed in 62.4% of patients with a primary GIST ≥2 cm or a metastatic GIST (721/1,156). The mutation analysis rate in reference centers was significantly higher than in non-reference centers (86.4% and 39.3%, resp., *p* < 0.01). In patients with a high-risk GIST or metastatic GIST, this difference was less pronounced (96.9% vs. 87.6%, *p* < 0.01).

### Localized GIST

#### Referral patterns according to the AFIP-Miettinen risk

**Figure 2:**
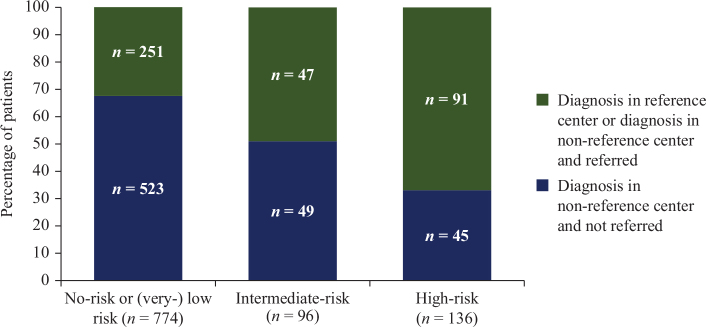
Referral patterns according to the AFIP-Miettinen risk classification in patients treated with primary surgery (*n* = 1,006).

Of the 1,403 patients with localized disease, the AFIP-Miettinen risk classification could be determined for 1,006 patients (Figure 2, Supplementary Table S1). In the remaining patients, tumor size or mitotic rate was missing. Fourteen per cent of GISTs were classified as high-risk. The majority of high-risk patients (66.9%) were referred to a reference center (74/136) or diagnosed in a reference center (17/136). In contrast, the majority of patients with a lower-risk GIST (i.e. no-risk, very low-risk or low-risk GIST) were diagnosed in non-reference centers and not referred to a reference center.

#### Differences in surgical outcomes in patients with localized GIST

Of 1,221 patients with a localized GIST that underwent surgery, 31.7% received surgery in a reference center. In 858 patients and in 5 patients, surgery was performed with curative intent or palliative intent, respectively. For the remaining patients (*n* = 145), the intent of surgery was not specified or GIST was an incidental finding (*n* = 213). Resection margins were missing in 58 patients. No significant difference was seen in the rate of R1/R2 resections between patients operated in reference centers and in non-reference centers (7.9% [19/242] vs. 6.6% [37/558], *p* = 0.84).

Data on tumor rupture was missing in 25.8% (315/1,221). The rate of tumor rupture did not significantly differ between patients operated in reference and in non-reference centers (3.0% [8/270] vs. 2.7% [17/636], *p* > 0.90).

#### Differences in systemic treatment in patients with localized GIST

Overall, in the timeframe of 6–9 months after GIST diagnosis, 27/136 high-risk patients did not receive adjuvant therapy, of which 11 were excluded from the analysis because of the presence of a non-imatinib sensitive mutation (*PDGFRA D842V* mutation [6/11], *KIT* and *PDGFRA wild-type* GIST [5/11]). Of the 125 high-risk patients, 77/86 (89.5%) of patients in reference centers and 32/39 (82.0%) patients in non-reference centers received imatinib in the (neo)adjuvant setting (*p* < 0.01). Of 96 intermediate-risk patients, 19/47 (40.4%) and 10/49 (20.4%) received imatinib in reference and in non-reference centers, respectively (*p* = 0.09).

#### Overall survival in patients with a high-risk GIST

**Figure 3:**
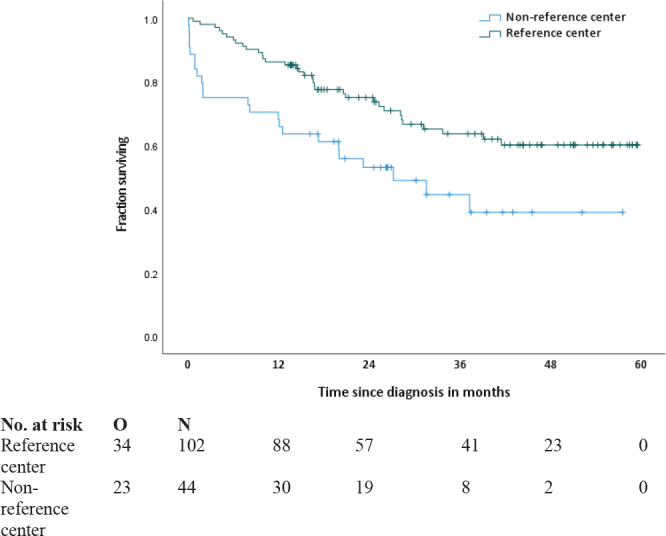
Overall survival in patients with high-risk GIST*. * Median OS was not reached.

Overall survival was analysed in the subgroup of patients with a high-risk GIST (*n* = 136, Figure 3). Due to the limited number of events (*n* = 11) and the short follow-up time (reference centers: median 33.3 months, range 5.4–60.0; non-reference centers: median 35.9 months, range 2.1–58.6), median OS was not reached.

### Metastatic GIST

#### Referral patterns in patients with metastatic GIST

The majority of metastatic GIST patients were diagnosed in non-reference centers (91.8%, [134/146]). Of these, 90 patients (67.2%) were subsequently referred to a reference center, whereas 44 patients (32.8%) were not referred to a reference center upon GIST diagnosis (Figure 1). Twelve metastatic GIST patients (8.2%) were diagnosed in a reference center upfront.

#### Treatment differences in patients with metastatic GIST

Overall, in 21/146 patients with metastatic GIST, systemic therapy was not started within 6–9 months after GIST diagnosis. Patients in non-reference centers were significantly less often treated with systemic treatment compared to patients in reference centers (65.9% vs. 94.1%, *p* < 0.01).

#### Overall survival in patients with metastatic GIST

Median follow-up time was 26.4 months (range <1–59.6 months) and 20.0 months (range <1–57.6 months) for patients in reference centers and in non-reference centers, respectively.

**Figure 4:**
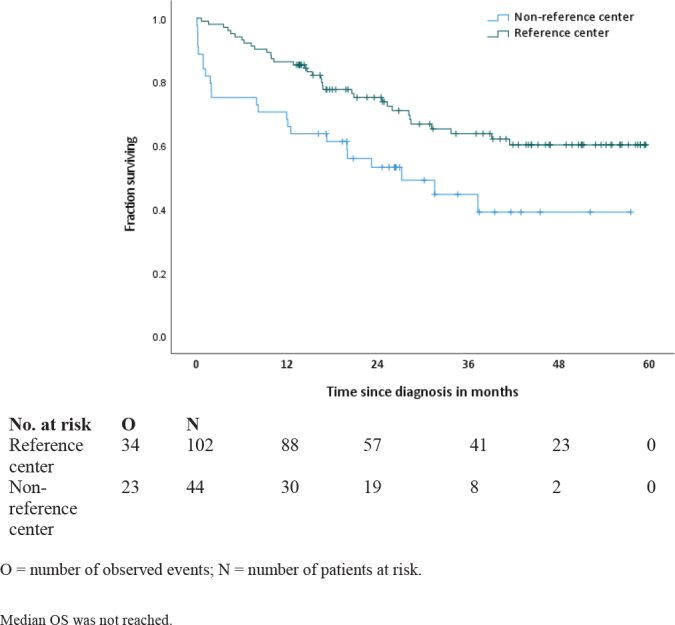
Overall survival in patients with metastatic GIST*. * Median OS was not reached.

Median OS for patients in reference centers was not reached (Figure 4). Median OS for patients in non-reference centers was 27.2 months (95% CI: 10.9–43.5 months).

## Discussion

This nationwide study analyzed data of a large cohort of 1550 GIST patients in the Netherlands. The majority of Dutch GIST patients were diagnosed in a non-reference center, and only one third of these patients was referred to a GIST reference center upon diagnosis. The referral rates for patients with a high-risk GIST (62.2%) and metastatic GIST (67.2%), respectively, illustrate that complex patients were more likely to be referred to a reference center. The high rates observed for mutation analysis and systemic treatment in high-risk GIST patients demonstrate that guideline adherence is high, both in reference centers and in non-reference centers. However, in a significant number of metastatic GIST patients in non-reference centers, systemic treatment was not started.

The higher age observed in patients in non-reference centers suggests that age could play a role when determining whether or not to refer a patient. Furthermore, in a substantial number of cases, GIST was an incidental finding (e.g. during surgery for another reason). In some cases, patient preference or ineligibility for treatment might have been reasons not to refer patients. However, these variables were not registered.

Previous research showed that centralizing treatment of sarcoma patients in reference centers leads to improved surgical outcomes and survival [10, 11]. In this study, only one third of surgeries for a localized GIST was performed in reference centers. This low number might be attributed to incidental GISTs, discovered during a procedure (e.g. gastroscopy) or surgery for another reason.

The R1/R2 resection rate (7.0%) and the rate of tumor rupture (2.8%) in this study were lower than reported in previous studies, and no significant differences were observed between patients operated on in reference centers and non-reference centers [12, 13]. However, GIST tumors in reference centers were significantly larger and significantly more often non-gastric, which could have led to different surgical procedures including multi-organ resections, making a comparison more difficult [14–16]. Furthermore, the rate of tumor rupture might be underestimated as data on tumor rupture was missing in 25.8% of cases. Regarding resection margins, exploring the rate of R1/R2 resection margins in the subgroup of high-risk GIST patients would have been interesting. Unfortunately, with only 136 high-risk patients, conducting this subgroup analysis is deemed not meaningful.

Since mutations are related both to prognosis and efficacy of treatment, the ESMO guidelines recommend mutation analysis in all patients with a GIST >2 cm [17]. In a previous Dutch study, mutation analysis was performed in only 33.9% of Dutch GIST patients that underwent a resection [7]. In the current study, mutation analysis was performed in 62.4% of patients with a GIST ≥2 cm. In high-risk and metastatic GIST patients, high testing rates of more than 87% were observed which is a big improvement since the start of mutation testing. A study conducted in the US between 2010 and 2015 showed lower mutation analysis rates of 26.7% in patients with localized GIST [18]. Knowing the mutation status is relevant in patients with an indication for systemic treatment as potential toxic treatments will only be given to patients with treatment sensitive mutations (for instance imatinib in case of *KIT* mutation and avapritinib in *PDGFRA D842V* mutations). Another study conducted in the US showed that mutation analysis was only performed in 40.7% of patients receiving (neo)adjuvant treatment [19]. In the same study, a physician survey revealed that physicians required more familiarity with the NCCN guidelines to know when and who to test.

In March 2011, adjuvant treatment with imatinib was officially implemented in clinical practice in the Netherlands for high-risk GIST patients. Farag et al. demonstrated in 2017 that 61% of Dutch high-risk GIST patients treated in a reference center with a diagnosis between 2011 and 2016 received adjuvant imatinib [20]. Similar results were reported in a Japanese study where 81% of high-risk patients received adjuvant therapy [21, 22]. In North-American reports ranging from 2004 to 2012, the percentage of high-risk patients treated with adjuvant therapy was lower, ranging between 18% and 58% [22–25]. In our study, the percentage of high risk patients receiving (neo)adjuvant imatinib increased to 82.0% in non-reference centers and 89.5% in reference centers. This suggests that over time, guideline adherence has improved in the Netherlands.

Our current study also shows that in reference centers, nearly all patients with a metastatic GIST received systemic therapy. In non-reference centers, despite a high mutation analysis rate, treatment rates for metastatic GIST patients were significantly lower (65.9%). Potential reasons for not receiving systemic therapy could be a difference in age, as high-risk and metastatic GIST patients in non-reference centers were significantly older (70.0 years vs. 65.0 years), which was also demonstrated in the study of Farag et al. [20] but could also be related to performance status, comorbidity or patient preference which were all not reported. Identifying the rationale for not starting systemic treatment in these patients could contribute to improving treatment patterns.

This study was limited by the retrospective study design and the design of both registries. Mitotic rate was missing in some patients, hence AFIP-Miettinen risk classification could not always be determined. As clinical variables were registered only up to 6–9 months after diagnosis, changes in therapy or relapse rate after that period of time are missing and were therefore not studied, and only patients with synchronic or early metastatic disease were included. Additionally, it would have been interesting to identify those patients that were never referred, not even after the 6–9 months interval, and evaluate survival outcomes in this particular subgroup. Regarding referral rates, only referral upon diagnosis was described. If a patient was referred to a reference center later on, this was not documented. In some cases, patients are only virtually discussed in the MTB of a reference center without being physically present in the hospital. As this type of referral was not registered in the NCR dataset, this causes an underestimation of the referral rate. Furthermore, documentation of these virtual MTB discussions could have given relevant information of reasons for non-referral. Due to the relatively short median follow-up time (34.1 months for high-risk patients; 14.7 months for patients with metastatic GIST), median OS was not reached.

To our knowledge, this is the first study describing referral patterns in GIST patients combined with detailed patient and tumor characteristics. This study is larger than most studies based on national GIST registries and gives an overview of patient and tumor characteristics with a complete national coverage.

## Conclusion

In conclusion, in the Netherlands more complex patients are more likely to be referred to a reference center, and in the vast majority of patients with a high-risk GIST, mutation analysis was performed and systemic treatment was started, both in reference and in non-reference centers. In non-reference centers, mutation analysis and systemic treatment were less common practice for all patients. Therefore, optimisation of referral patterns could benefit patients. The frequency of R0 resections and tumor rupture was similar in reference and non-reference centers. The reasons for not performing mutation analysis or not starting systemic treatment in non-reference centers are subject to further research.

## Supplementary Material

Referral patterns of GIST patients: data from a nationwide study

## Data Availability

The data that support the findings of this study are available from IKNL and palga. Restrictions apply to the availability of these data, which were used under license for this study. Data are only available with the permission of IKNL and palga.

## References

[1] Miettinen M, Lasota J. Gastrointestinal stromal tumors: review on morphology, molecular pathology, prognosis, and differential diagnosis. Arch Pathol Lab Med. 2006;130(10):1466–78. 10.5858/2006-130-1466-GSTROM 17090188

[2] Søreide K, Sandvik OM, Søreide JA, Giljaca V, Jureckova A, Bulusu VR. Global epidemiology of gastrointestinal stromal tumours (GIST): a systematic review of population-based cohort studies. Cancer Epidemiol. 2016;40:39–46. 10.1016/j.canep.2015.10.031 26618334

[3] Federatie medisch Specialisten. Normeringsrapport van SONCOS 2022. Available from: https://demedischspecialist.nl/normeringsrapport-van-soncos [cited 02-05-2023]

[4] Casali PG, Blay JY, Abecassis N, et al. Gastrointestinal stromal tumours: ESMO-EURACAN-GENTURIS clinical practice guidelines for diagnosis, treatment and follow-up. Ann Oncol. 2022;33(1):20–33. 34560242 10.1016/j.annonc.2021.09.005

[5] Joensuu H, Vehtari A, Riihimäki J, et al. Risk of recurrence of gastrointestinal stromal tumour after surgery: an analysis of pooled population-based cohorts. Lancet Oncol. 2012;13(3):265–74. 10.1016/S1470-2045(11)70299-6 22153892

[6] Bauer S, George S, von Mehren M, Heinrich MC. Early and next-generation KIT/PDGFRA kinase inhibitors and the future of treatment for advanced gastrointestinal stromal tumor. Front Oncol. 2021;11:672500. 10.3389/fonc.2021.672500 34322383 PMC8313277

[7] Steeghs EMP, Gelderblom H, Ho VKY, et al. Nationwide evaluation of mutation-tailored treatment of gastrointestinal stromal tumors in daily clinical practice. Gastric Cancer. 2021;24(5):990–1002. 33909171 10.1007/s10120-021-01190-9 PMC8338807

[8] Verschoor AJ, Bovée J, Overbeek LIH, Hogendoorn PCW, Gelderblom H. The incidence, mutational status, risk classification and referral pattern of gastro-intestinal stromal tumours in the Netherlands: a nationwide pathology registry (PALGA) study. Virchows Arch. 2018;472(2):221–9. 10.1007/s00428-017-2285-x 29308530 PMC5856869

[9] Casparie M, Tiebosch AT, Burger G, et al. Pathology databanking and biobanking in The Netherlands, a central role for PALGA, the nationwide histopathology and cytopathology data network and archive. Cell Oncol. 2007;29(1):19–24. 10.1155/2007/971816 17429138 PMC4618410

[10] Blay JY, Soibinet P, Penel N, et al. Improved survival using specialized multidisciplinary board in sarcoma patients. Ann Oncol. 2017;28(11):2852–9. 10.1093/annonc/mdx484 29117335 PMC5834019

[11] Blay JY, Honoré C, Stoeckle E, et al. Surgery in reference centers improves survival of sarcoma patients: a nationwide study. Ann Oncol. 2019;30(7):1143–53. 10.1093/annonc/mdz124 31081028 PMC6637376

[12] Gronchi A, Bonvalot S, Poveda Velasco A, et al. Quality of surgery and outcome in localized gastrointestinal stromal tumors treated within an international intergroup randomized clinical trial of adjuvant imatinib. JAMA Surg. 2020;155(6):e200397. 10.1001/jamasurg.2020.0397 32236507 PMC7113837

[13] Hølmebakk T, Bjerkehagen B, Hompland I, Stoldt S, Boye K. Relationship between R1 resection, tumour rupture and recurrence in resected gastrointestinal stromal tumour. Br J Surg. 2019;106(4):419–26. 10.1002/bjs.11027 30507040

[14] Blum MG, Bilimoria KY, Wayne JD, de Hoyos AL, Talamonti MS, Adley B. Surgical considerations for the management and resection of esophageal gastrointestinal stromal tumors. AnnThorac Surg. 2007;84(5):1717–23. 10.1016/j.athoracsur.2007.05.071 17954092

[15] Kameyama H, Kanda T, Tajima Y, et al. Management of rectal gastrointestinal stromal tumor. Transl Gastroenterol Hepatol. 2018;3:8. 10.21037/tgh.2018.01.08 29552659 PMC5847932

[16] Ijzerman NS, Mohammadi M, Tzanis D, et al. Quality of treatment and surgical approach for rectal gastrointestinal stromal tumour (GIST) in a large European cohort. Eur J Surg Oncol. 2020;46(6):1124–30. 10.1016/j.ejso.2020.02.033 32224070

[17] Joensuu H, Wardelmann E, Sihto H, et al. Effect of KIT and PDGFRA mutations on survival in patients with gastrointestinal stromal tumors treated with adjuvant imatinib: an exploratory analysis of a randomized clinical trial. JAMA Oncol. 2017;3(5):602–9. 10.1001/jamaoncol.2016.5751 28334365 PMC5470395

[18] Florindez J, Trent J. Low frequency of mutation testing in the United States: an analysis of 3866 GIST patients. Am J Clin Oncol. 2020;43(4):270–8. 10.1097/COC.0000000000000659 31904710

[19] Bartholomew AJ, Dohnalek H, Prins PA, et al. Underuse of exon mutational analysis for gastrointestinal stromal tumors. J Surg Res. 2018;231:43–8. 10.1016/j.jss.2018.05.014 30278964

[20] Farag S, van Coevorden F, Sneekes E, et al. Elderly patients with gastrointestinal stromal tumour (GIST) receive less treatment irrespective of performance score or comorbidity – a retrospective multicentre study in a large cohort of GIST patients. Eur J Cancer. 2017;86:318–25. 10.1016/j.ejca.2017.09.017 29073582

[21] Nishida T, Sakai Y, Takagi M, et al. Adherence to the guidelines and the pathological diagnosis of high-risk gastrointestinal stromal tumors in the real world. Gastric Cancer. 2020;23(1):118–25. 10.1007/s10120-019-00966-4 31041650 PMC6942594

[22] Bischof DA, Dodson R, Jimenez MC, et al. Adherence to guidelines for adjuvant imatinib therapy for GIST: a multi-institutional analysis. J Gastrointest Surg. 2015;19(6):1022–8. 10.1007/s11605-015-2782-7 25731828

[23] Pisters PWT, Blanke CD, von Mehren M, et al. A USA registry of gastrointestinal stromal tumor patients: changes in practice over time and differences between community and academic practices. Ann Oncol. 2011;22(11):2523–9. 10.1093/annonc/mdq773 21464155

[24] Bilimoria KY, Wayne JD, Merkow RP, et al. Incorporation of adjuvant therapy into the multimodality management of gastrointestinal stromal tumors of the stomach in the United States. Ann Surg Oncol. 2012;19(1):184–91. 10.1245/s10434-011-1842-9 21725688

[25] Bischof DA, Kim Y, Blazer DG, 3rd et al. Surgical management of advanced gastrointestinal stromal tumors: an international multi-institutional analysis of 158 patients. J Am Coll Surg. 2014;219(3):439–49. 10.1016/j.jamcollsurg.2014.02.037 25065359

