# Supplementary material for: Referral patterns of GIST patients: data from a nationwide study
Source: Acta Oncol. 2024 Feb 14;63:23722. doi: 10.2340/1651-226X.2024.23722 (PMC11332507; doi:10.2340/1651-226X.2024.23722)
Supplement: Referral patterns of GIST patients: data from a nationwide study [file AO-63-23722-s1.pdf]

Supplementary material has been published as submitted. It has not been copyedited or typeset by Acta Oncologica.

Supplementary Table S1. AFIP-Miettinen classification in patients with surgery for localized GIST (n=1006)

| | **No-risk** (n=275) | **Very low risk** (n=270) | **Low-risk** (n=229) | **Intermediate-risk** (n=96) | **High-risk** (n=136) |
|---|---|---|---|---|---|
| Diagnosis in non-reference center and not referred | 199 (72.4) | 182 (67.4) | 142 (62.0) | 49 (51.0) | 45 (33.1) |
| Diagnosis in non-reference center and referred | 18 (6.5) | 65 (24.1) | 66 (28.8) | 42 (43.8) | 74 (54.4) |
| Diagnosis in reference center | 58 (21.1) | 23 (8.5) | 21 (9.2) | 5 (5.2) | 17 (12.5) |
